# Ventral striatal dysfunction in cocaine dependence – difference mapping for subregional resting state functional connectivity

**DOI:** 10.1038/s41398-018-0164-0

**Published:** 2018-06-18

**Authors:** Sheng Zhang, Chiang-Shan R. Li

**Affiliations:** 1 Department of Psychiatry, Yale University, New Haven, CT 06519 USA; 2 Department of Neuroscience, Yale University, New Haven, CT 06520 USA; 3 Interdepartmental Neuroscience Program, Yale University, New Haven, CT 06520 USA

## Abstract

Research of dopaminergic deficits has focused on the ventral striatum (VS) with many studies elucidating altered resting state functional connectivity (rsFC) in individuals with cocaine dependence (CD). The VS comprises functional subregions and delineation of subregional changes in rsFC requires careful consideration of the differences between addicted and healthy populations. In the current study, we parcellated the VS using whole-brain rsFC differences between CD and non-drug-using controls (HC). Voxels with similar rsFC changes formed functional clusters. The results showed that the VS was divided into 3 subclusters, in the area of the dorsal-anterior VS (daVS), dorsal posterior VS (dpVS), and ventral VS (vVS), each in association with different patterns of rsFC. The three subregions shared reduced rsFC with bilateral hippocampal/parahippocampal gyri (HG/PHG) but also showed distinct changes, including reduced vVS rsFC with ventromedial prefrontal cortex (vmPFC) and increased daVS rsFC with visual cortex in CD as compared to HC. Across CD, daVS visual cortical connectivity was positively correlated with amount of prior-month cocaine use and cocaine craving, and vVS vmPFC connectivity was negatively correlated with the extent of depression and anxiety. These findings suggest a distinct pattern of altered VS subregional rsFC in cocaine dependence, and some of the changes have eluded analyses using the whole VS as a seed region. The findings may provide new insight to delineating VS circuit deficits in cocaine dependence and provide an alternative analytical framework to address functional dysconnectivity in other mental illnesses.

## Introduction

Abundant research of dopaminergic deficits in addiction has focused on the ventral striatum (VS)^1^. The VS receives projections from the dopaminergic midbrain and processes reward-related stimuli, including those associated with drugs of abuse^1,2^. For instance, positron emission tomography (PET) imaging studies showed that the extracellular concentration of dopamine in the VS increased with drug administration, in link with experienced euphoria^3^. As with dopamine receptor blockade, dopamine-specific VS lesioning disrupted self-administration of intravenous cocaine^4^. In a study combining PET and magnetic resonance imaging, dopamine D2 receptor availability in the VS was significantly related to the blood oxygenation level-dependent (BOLD) responses to monetary reward in individuals with cocaine dependence (CD)^5^. Other studies showed that the VS responded to both acute cocaine administration and cue-elicited craving^6–9^.

Individuals with cocaine addiction demonstrated altered functional connectivities of the VS^10–19^, including reduced resting state functional connectivity (rsFC) between the VS and precuneus/posterior cingulate cortex^17^. These studies employed various templates of the VS as the seed region. On the other hand, the inferior and superior VS each showed connectivity with the medial and lateral orbitofrontal cortex^20^ and the ventral tegmental area (VTA) showed reduced rsFC with right dorsal posterior VS but not with the entire VS in CD^12^. These findings suggest distinct VS subregional connectivities, which may differ between CD and non-drug using healthy controls (HC) in specific patterns. Importantly, as a result of the chronic influence of cocaine, VS functional subregions may not follow the same boundary in CD as in HC. It would require a new approach to investigate how VS connectivities are altered in cocaine addiction or other conditions that implicate dopaminergic dysfunction.

Functional subdivisions of a brain region can be parcellated via unique patterns of connectivities^21^. Specifically, low-frequency BOLD signal fluctuations reflect connectivity between functionally related brain regions, which characterizes the intrinsic organization of the brain^22^. As regions with similar functionality tend to be correlated in their spontaneous BOLD activity, investigators described subareal functional boundaries for many cortical and subcortical structures. For instance, our previous study showed that the precuneus could be divided into dorsal-anterior, dosal-posterior, and ventral subregions by rsFC, each with distinct and sometimes an opposing pattern of regional connectivities^23^. It is argued that although not necessarily in agreement with anatomical demarcations, these functional boundaries may better explain regional activations to task challenges and disambiguate seeming discrepancies across studies^24^.

The current work aimed to address VS subareal rsFC in cocaine addiction. Considering that altered connectivity patterns of the VS may influence functional clustering of the subregions, we employed the connectivity differences between CD and HC to parcellate the VS. Thus, voxels with a similar difference pattern will be grouped into one cluster and a comparison of whole-brain connectivity of these clusters would reflect how CD and HC differ in VS connectivity. We also compared these results with those obtained with the entire VS as a seed region and examined how these regional connectivity differences relate to clinical characteristics.

## Materials and methods

### Subjects, informed consent, and assessment

Sixty-six recently abstinent participants (44 men) with cocaine dependence (CD) and 66 age- and gender-matched healthy control (HC) subjects (36 men) participated in the study (Supplementary Table 1). CD met criteria for current cocaine dependence, as diagnosed by the Structured Clinical Interview for DSM-IV^25^. Recent cocaine use was confirmed by urine toxicology screens. CD were drug-free while staying in an inpatient unit prior to the current fMRI study. All subjects were physically healthy with no major medical illnesses or current use of prescription medications. None reported having a history of head injury or neurological illness. Other exclusion criteria included dependence on another psychoactive substance (except nicotine) and current or past history of psychotic disorders. Individuals with current depressive or anxiety symptoms requiring treatment or currently being treated for these symptoms were excluded as well. The Human Investigation committee at Yale University School of Medicine approved all study procedures, and all subjects signed an informed consent prior to study participation.

CD’s were assessed with the Beck Depression Inventory^26^ and the State-Trait Anxiety Inventory^27^ at admission. The mean ( ± SD) BDI (9.5 ± 8.1) and STAI state (33.8 ± 11.0) and trait (38.3 ± 9.8) scores were within the range reported previously for individuals with cocaine dependence^28,29^. Cocaine craving was assessed with the cocaine craving questionnaire, brief version (CCQ-Brief), for all participants every two to three days^30^. The CCQ-Brief is a 10-item questionnaire, abbreviated from the CCQ-Now. It is highly correlated with the CCQ-Now and other cocaine craving measures^30^. Each item was rated on a scale from 1 to 7, with a higher total score (ranging from 10 to 70) indicating greater craving. CD showed a CCQ score of 23.8 ± 10.4 across all assessments.

### Imaging protocol

Conventional T1-weighted spin echo sagittal anatomical images were acquired for slice localization using a 3 T scanner (Siemens Trio). Anatomical images of the functional slice locations were next obtained with spin echo imaging in the axial plane parallel to the AC–PC line with TR = 300 ms, TE = 2.5 ms, bandwidth = 300 Hz/pixel, flip angle = 60°, field of view = 220 × 220 mm, matrix = 256 × 256, 32 slices with slice thickness = 4 mm and no gap. Functional, blood oxygenation level-dependent (BOLD) signals were then acquired with a single-shot gradient echo echoplanar imaging (EPI) sequence during a resting state (one scan per participant; duration: 10 minutes; eyes closed). Thirty-two axial slices parallel to the AC–PC line covering the whole brain were acquired with TR = 2000 ms, TE = 25 ms, bandwidth = 2004 Hz/pixel, flip angle = 85°, field of view = 220 × 220 mm, matrix = 64 × 64, 32 slices with slice thickness = 4 mm and no gap.

### Imaging data preprocessing

**Fig. 1:**
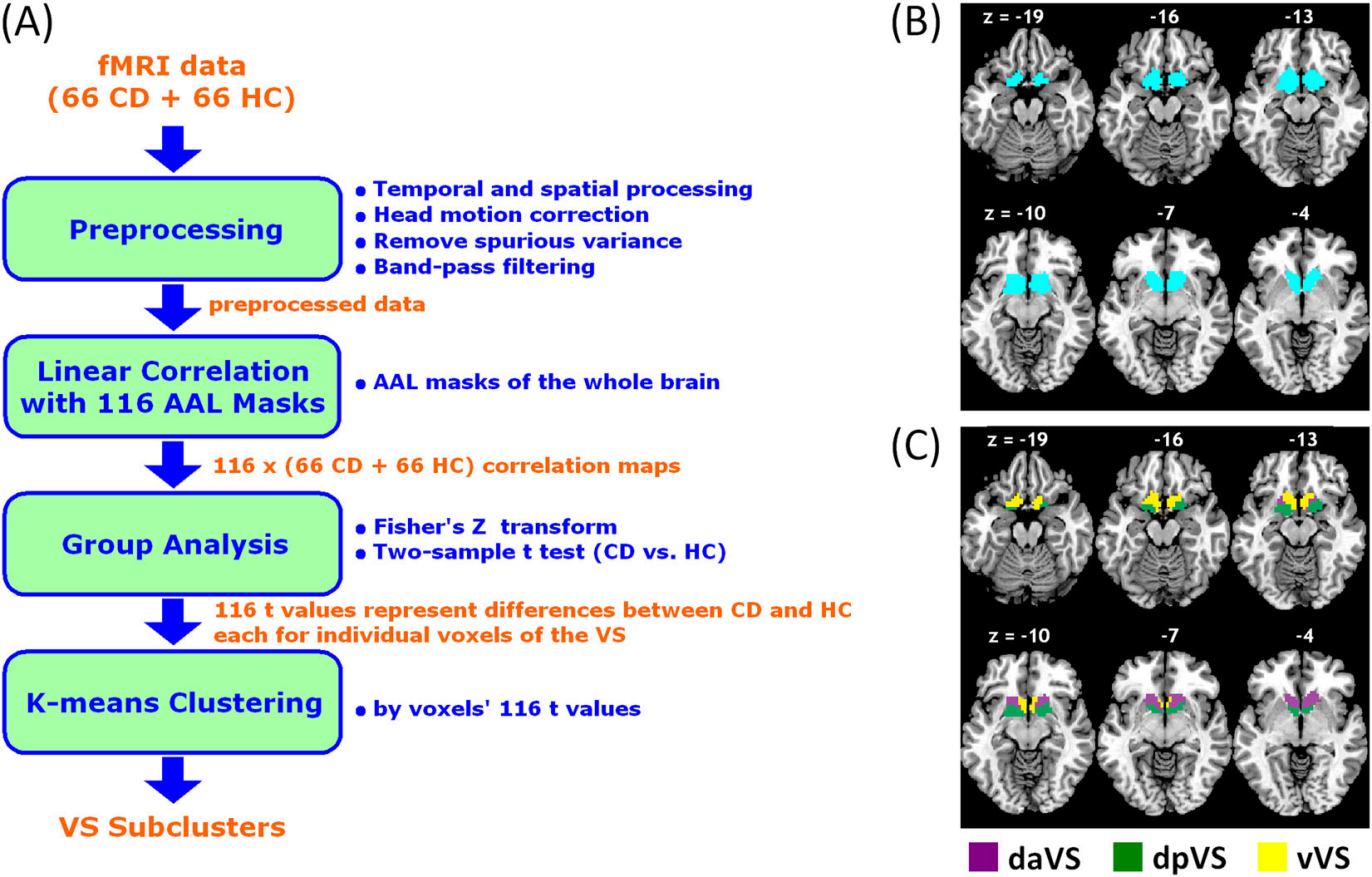
**Data analytic procedures and subregions of the ventral striatum.** (**a**) A flow chart of data analysis. (**b**) Seed region: the ventral striatum (VS). (**c**) K-means clustering segments the VS based on connectivity differences between CD and HC of individual voxels within the region. Three subclusters were represented with different colors as dorsal-anterior VS (daVS), dorsal posterior VS (dpVS), and ventral VS (vVS)

Figure 1a illustrates the step by step procedures in data analysis. The data were processed with Statistical Parametric Mapping (SPM8, Wellcome Department of Imaging Neuroscience, University College London, U.K.). Images from the first five TRs at the beginning of each session were discarded. Images of each individual subject were first realigned (motion corrected) and corrected for slice timing. A mean functional image volume was constructed from the realigned image volumes. These mean images were co-registered with the high-resolution structural image and segmented for normalization with affine registration followed by nonlinear transformation. The normalization parameters determined for the structure volume were then applied to the corresponding functional image volumes for each subject. Finally, the images were smoothed with a Gaussian kernel of 4 mm at Full Width at Half Maximum.

Additional preprocessing was applied to reduce spurious BOLD variances that were unlikely to reflect neuronal activity. The sources of spurious variance were removed through linear regression by including the signal from the ventricular system, white matter, and whole brain, in addition to the six parameters obtained by rigid body head motion correction^22,31^. First-order derivatives of the whole brain, ventricular and white matter signals were also included in the regression.

Cordes and colleagues suggested that BOLD fluctuations below a frequency of 0.1 Hz contribute to regionally specific BOLD correlations^32^. Thus, we applied a temporal band-pass filter (0.009 Hz < *f* < 0.08 Hz) to the time course in order to obtain low-frequency fluctuations, as in previous studies^22^.

### Head motion

As extensively investigated in previous study, micro head motion (>0.1 mm) is an important source of spurious correlations in resting state functional connectivity analysis^33^. Therefore, we applied a “scrubbing” method proposed by Power and colleagues^34^ and successfully applied in previous studies^35^ to remove time points affected by head motions, prior to temporal band-pass filtering. Briefly, for every time point *t*, we computed the framewise displacement given by${\mathrm{FD}}\left( {{t}} \right) = \left| {\Delta {d}_{x}\left( {t} \right)} \right| + \left| {\Delta {d}_{y}\left( {t} \right)} \right| + \left| {\Delta {d}_{z}\left( {t} \right)} \right| + \left| {\Delta {\mathrm{\alpha }}\left( {t} \right)} \right| + \left| {\Delta {\mathrm{\beta }}\left( {t} \right)} \right| + |\Delta {\mathrm{\gamma }}\left( {t} \right)|$, where (*d*_*x*_, *d*_*y*_, *d*_*z*_) and (*α*, *β*, *γ*) are the translational and rotational movements, respectively^34^. The second head movement metric was the root mean square variance (DVARS) of the differences in % BOLD intensity *I*(*t*) between consecutive time points across brain voxels, computed as: ${\mathrm{DVARS}}\left( {t} \right) = \sqrt {|{I}\left( {t} \right) - {I}({t} - 1)|^2}$, where the brackets indicate the mean across brain voxels. Finally, to compute each subject’s correlation map, we removed every time point with FD(*t*) > 0.5 mm or DVARS(*t*) > 0.5%^34,35^. On average, 1% of the time points were removed across subjects. CD and HC did not differ in either the mean FD (*p* = 0.76) or DVARS (*p* = 0.57).

### Parcelation of the VS based on functional connectivity differences between CD and HC

As with our previous studies^36,37^, we used a ventral striatum (VS) mask (Fig. 1b**)** generated by cytoarchitectonic and topographical criteria^38^. We employed 116 anatomical masks from the Automated Anatomical Labeling or AAL Atlas based on a Montreal Neurological Institute (MNI) template^39^. We averaged the BOLD time courses of voxels within each of the 116 regions and computed the correlation coefficient between the averaged time course of each AAL region and the time courses of each individual voxel of the VS for individual subjects. To assess and compare the resting state “correlograms (correlation matrices),” we converted these correlation matrices, which were not normally distributed, to z score maps by Fisher’s z transform:${\mathrm{z}} = 0.5{\mathrm{log}}_e[\frac{{1 + r}}{{1 - r}}]$. The z maps were used in group, random effect analyses.

A two-sample *t* test was first applied to the “z maps” to compare 66 CD with 66 HC for each of the 116 correlograms. Voxels within the VS mask were subject to rsFC based segmentation, with each voxel represented by 116 *t* values of the two-sample *t* test. A K-means algorithm was applied to cluster the voxels within the VS on the bases of the 116 *t* values.

As an unsupervised learning algorithm, K-means clustering classifies a given data set into an a-priori set of *K* clusters by minimizing an objective squared error function as shown in Eq. (1):.

1 $${J} = \mathop {\sum }\limits_{j = 1}^k \mathop {\sum }\limits_{i = 1}^n x_i^{(j)} - c_j^2$$

where $x_i^{(j)} - c_j^2$ is a distance measure between a data point $x_i^{(j)}$ and the cluster center $c_j$^40^. The algorithm was executed by:

1. *Placing K points into the space represented by the objects that are being clustered. These points represent initial group centroids*.
2. *Assigning each object to the group that has the closest centroid*.
3. *Recalculating the positions of the K centroids, when all objects have been assigned*.
4. *Repeating Steps 2 and 3 until the centroids no longer move. This produces a separation of the objects into groups from which the metric to be minimized can be calculated*.

In order to determine the optimal number of clusters that best described the data set, we used the Bayesian Information Criterion (BIC), which is widely used for model identification in time series and linear regression:

2 $${\mathrm{BIC}} = n\ln (\frac{{{\rm{RSS}}}}{n}) + k\ln (n)$$

where *n* is the number of observations (=116); *k* is the number of class; RSS is the residual sum of squares from the K-means model. Given any two clustering number *k’s*, the one with lower BIC value was preferred. Furthermore, because the K-means algorithm is sensitive to the initial, randomly selected cluster centers, we repeated this algorithm 1,000 times to alleviate the effect of the initial conditions. K was tested for values from 2 to 20.

For VS subregions as well as the whole VS, we computed the whole-brain connectivity maps each for CD and HC, and converted to z score maps. In second level analysis, we performed one-sample *t* test each on the Z maps of those seeds for CD and HC groups and two-sample *t* test with age as covariate comparing the two groups. In addition, the VS and hypothalamus are reciprocally connected^2^. Both the VS and hypothalamus receive projections from the dopaminergic midbrain and are implicated in processing of reward-related stimuli, including those associated with drugs of abuse^2,41–44^. Examining the VS hypothalamus connectivity may provide useful information as to how this understudied circuit is impacted by cocaine addiction^3,42,45–47^. Thus, we also conducted a region of interest analysis, using small volume correction for a hypothalamus mask^48^.

## Results

### VS functional subclusters and connectivity differences between CD and HC

**Fig. 2:**
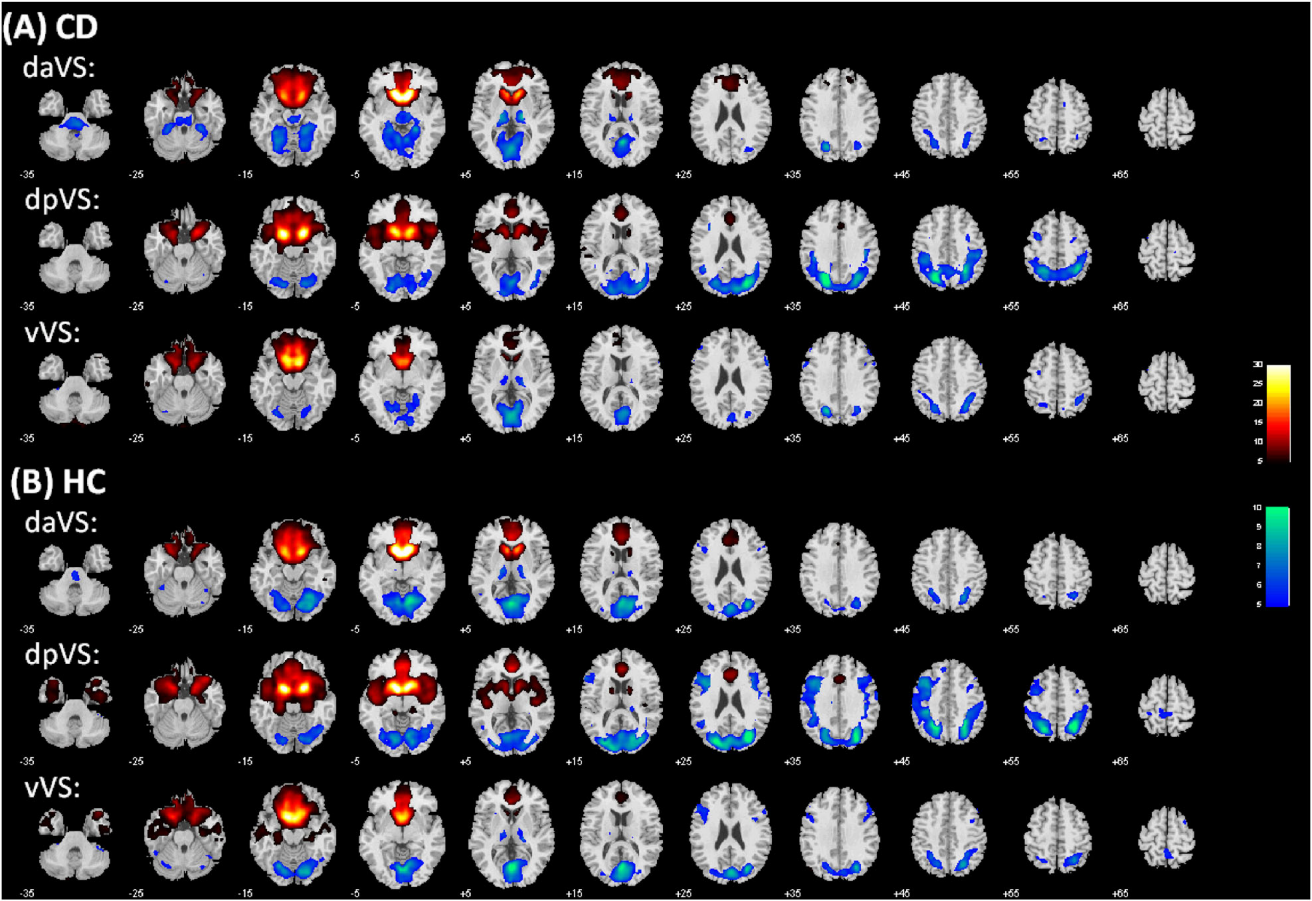
Ventral striatum subregional connectivity. One-sample *t* test results of functional connectivity maps of the dorsal anterior VS (daVS), dorsal posterior VS (dpVS), and ventral VS (vVS) each for (**a**) CD and (**b**) HC. *p* < 0.05, corrected for familywise error of multiple comparisons

The results of 1,000 runs of K-means clustering suggested an optimal cluster number of 3 according to the BIC (Supplementary Figure 1). Figure 1c showed the three clusters as dorsal anterior (daVS), dorsal posterior (dpVS), and ventral VS (vVS). Figure 2 showed the whole-brain connectivity maps of the three clusters each for CD and HC.

**Fig. 3:**
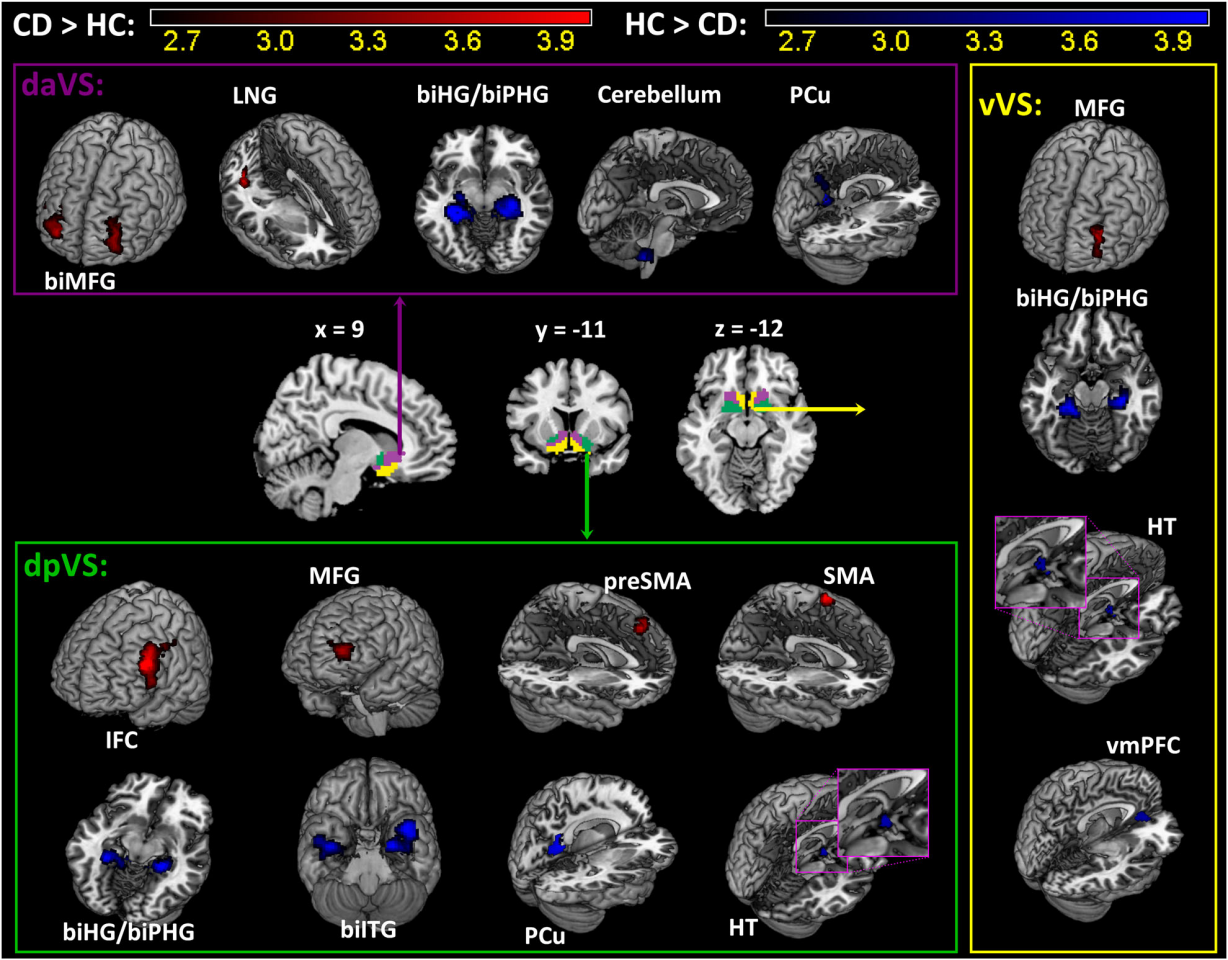
Altered functional connectivity in CD compared to HC is shown for each of the three subclusters of the VS. Warm color: CD > HC; cool color: HC > CD. Details can be found in Supplementary Table 2. LNG: Lingual gyrus; biHG: bilateral hippocampus; biPHG: bilateral parahippocampus; PCu: precuneus; IFC: inferior frontal cortex; MFG: middle frontal gyrus; SMA: supplementary motor area; preSMA: pre-supplementary motor area; biITG: bilateral inferior temporal gyrus; vmPFC: ventromedial prefrontal gyrus; HT: hypothalamus

We then examined differences in connectivity of each of the three subclusters in CD as compared to HC in a two-sample *t* test with age as a covariate at voxel *p* < 0.005, uncorrected, in combination with cluster *p* < 0.05, FWE corrected, on the basis of Gaussian random field theory as implemented in SPM. The results are shown in Fig. 3 and Supplementary Table 2. Compared to HC, CD showed increased left middle frontal gyrus (MFG) connectivity and decreased bilateral hippocampal/parahippocampal gyri (HG/PHG) connectivity for all three subclusters. The subclusters also showed distinct differences in connectivity between CD and HC. Both daVS and dpVS showed decreased rsFC with the precuneus, and both dpVS and vVS showed decreased rsFC with the hypothalamus, in CD as compared to HC. Further, the daVS showed increased rsFC with the right MFG and lingual gyrus, and decreased rsFC with the cerebellum, in CD as compared to HC. The dpVS showed increased rsFC with the left inferior frontal cortex (IFC), supplementary motor area (SMA), and pre-SMA, and decreased rsFC with the bilateral inferior temporal gyri (ITG). The vVS showed decreased rsFC with the ventromedial prefrontal cortex (vmPFC) in CD as compared to HC.

### Relationship to clinical characteristics

For CD, we examined whether these connectivity changes are related to clinical characteristics with a linear regression of the connectivity strength (z score) of each ROI against recent cocaine use (amount of cocaine use in the past month, grams), craving (CCQ score), the extent of depression (BDI score) and of anxiety (STAI state and trait score). Because of the 17 identified connectivity pairs and five clinical measures, we evaluated the results at a corrected *p* = 0.05 / (17 × 5) = 0.00059.

**Fig. 4:**
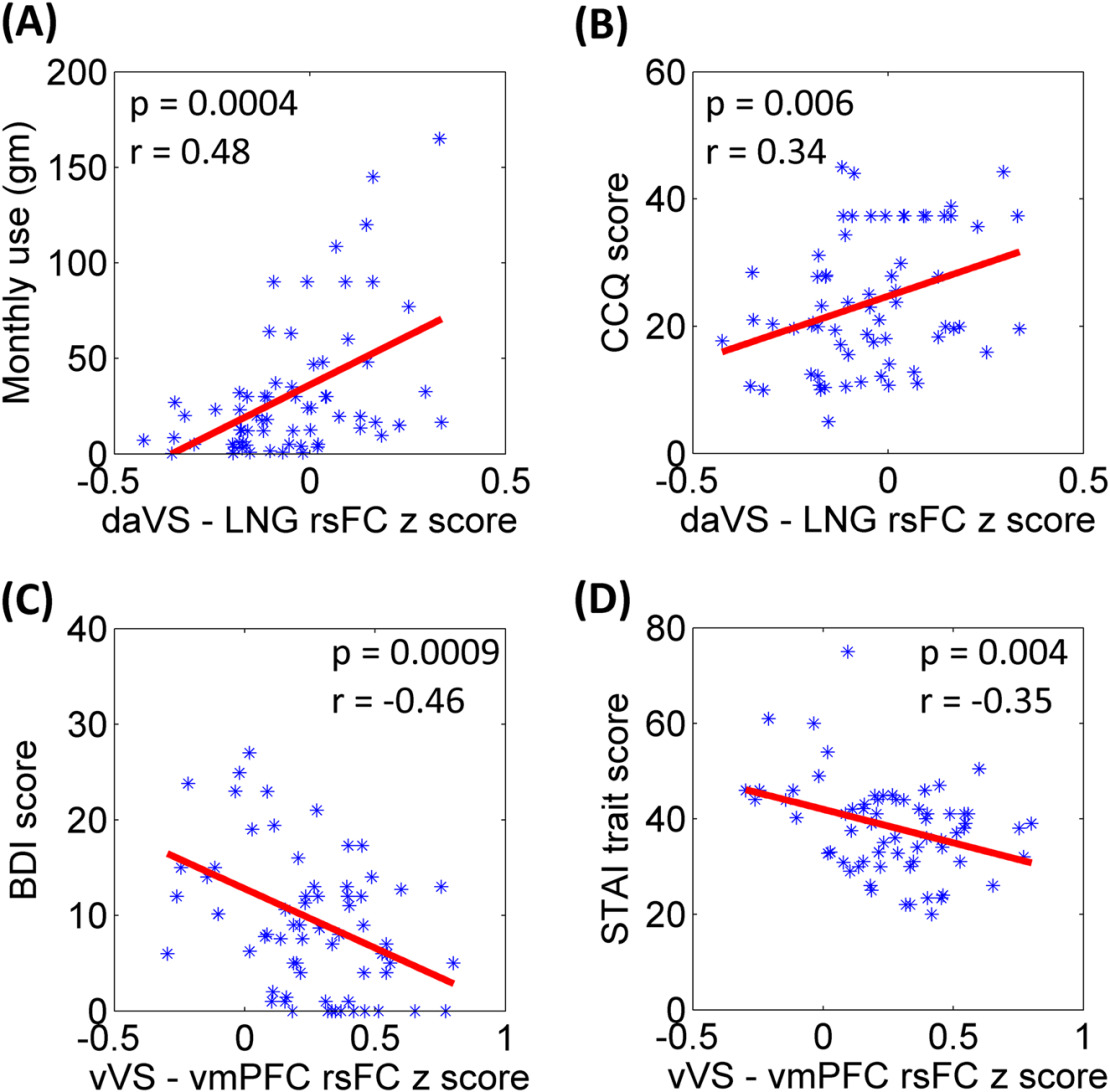
Correlation of VS connectivity with clinical measures. Dorsal anterior VS (daVS) connectivity with the lingual gyrus (LNG) was positively correlated with (**a**) the average monthly use of cocaine (grams) in the prior year and with (**b**) craving score as assessed by the Cocaine Craving Questionnaire (CCQ). Ventral VS (vVS) connectivity with the ventromedial prefrontal cortex (vmPFC) was negatively correlated with (**c**) depression score, as assessed by the Beck Depression Inventory (BDI) and with (**d**) trait anxiety score, as assessed by the Spielberger State Trait Anxiety Inventory (STAI). Correlations in (**b**) and (**d**) were significant only at a trend level, with correction for multiple comparisons

Across CD, the daVS and lingual gyrus connectivity strength was positively correlated with amount of cocaine use in the past month (*p* = 0.0004, *r* = 0.48), and, at a trend level, averaged CCQ score (*p* = 0.006, *r* = 0.34). The vVS–vmPFC connectivity was negatively correlated with the BDI score (*p* = 0.0009, *r* = −0.46) and, at a trend level, STAI trait score (*p* = 0.004, *r* = −0.35). Figure 4 summarizes these results.

### Connectivity of the whole VS

**Fig. 5:**
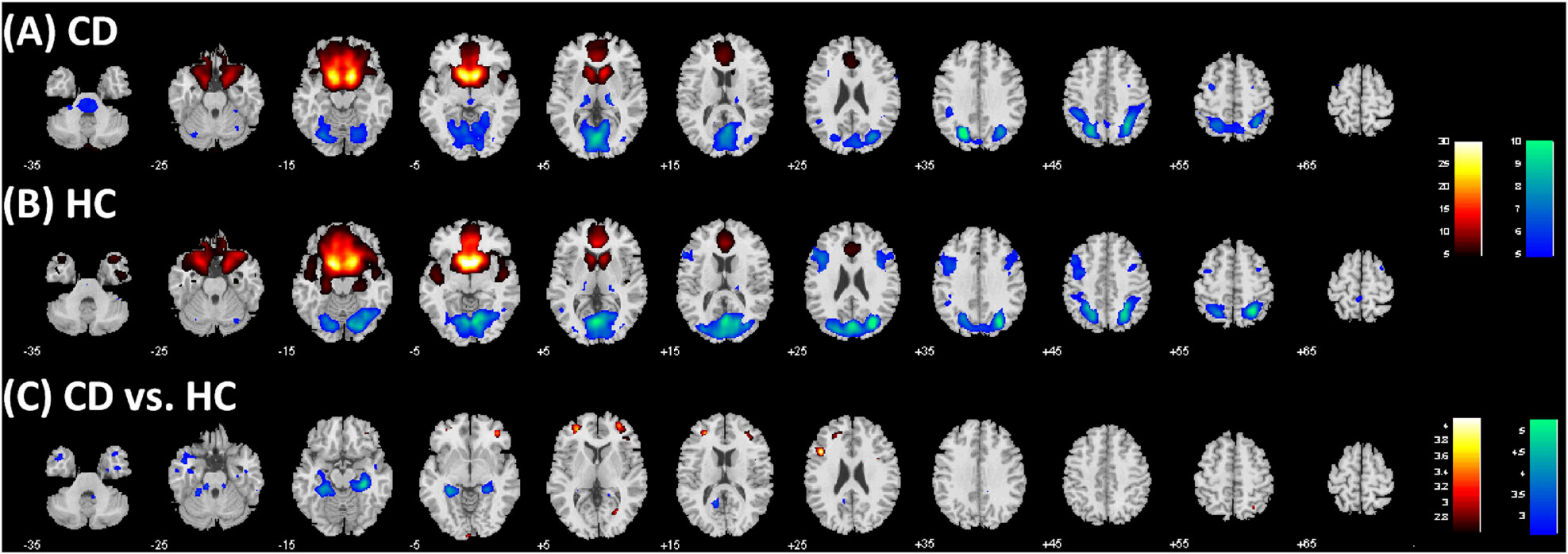
Ventral striatum connectivity in CD and HC. One sample t test of whole-brain functional connectivity with the entire VS as the seed each in (**a**) CD and (**b**) HC, p < 0.05 FWE; as well as (**c**) the two-sample t test results of CD vs. HC, p < 0.005 uncorrected

For comparison, we examined how CD and HC differed in rsFC with the whole VS as the seed. Figure 5 shows the one-sample t test results of whole-brain rsFC each for CD and HC as well as two-sample t test results of CD vs. HC. In both CD and HC, VS showed positive connectivity with the dorsal and ventral medial prefrontal cortex, caudate, and insula, and negative connectivity with the thalamus, inferior parietal gyrus, and visual cortex. Compared to HC, CD showed increased VS connectivity with the bilateral middle frontal gyri (*x* = −21, *y* = 38, *z* = 16, peak voxel *Z* = 3.84, volume = 5,832 mm^3^; *x* = 27, *y* = 32, *z* = 16, peak voxel *Z* = 3.49, volume = 4,833 mm^3^), decreased VS connectivity with the bilateral HG/PHG (*x* = −30, *y* = −34, *z* = −8, peak voxel *Z* = 4.64, volume = 11,016 mm^3^; *x* = 30, *y* = −28, *z* = −14, peak voxel *Z* = 5.02, volume = 8,775 mm^3^), at a threshold of voxel *p* < 0.005 uncorrected in combination with cluster-level *p* < 0.05, FWE corrected. At a relaxed threshold – voxel *p* < 0.005 uncorrected and AlphaSim *p* < 0.05 corrected-CD additionally showed increased VS connectivity with the left IFC (*x* = −42, *y* = 17, *z* = 25, peak voxel *Z* = 3.96, volume = 1,431 mm^3^) and decreased VS connectivity with the precuneus (*x* = −15, *y* = −49, *z* = 16, peak voxel *Z* = 4.18, volume = 3078 mm^3^).

We further examined whether these connectivity differences are related to clinical characteristics. The VS – precuneus connectivity was correlated with amount of cocaine use in the past month at *p* = 0.03, *r* = 0.27, and all other correlations were at *p*’s > 0.17, none of which were significant considering multiple comparisons.

## Discussion

We demonstrated that the VS could be separated into dorsal anterior (daVS), dorsal posterior (dpVS), and ventral (vVS) subregions on the basis of whole-brain resting state functional connectivity (rsFC) differences between CD and HC. These subregions reflect how the VS is functionally organized in terms of the differences between CD and HC in voxelwise connectivity. CD showed increased connectivity with the left middle frontal gyrus (MFG) and decreased connectivity in the bilateral hippocampal/parahippocampal gyri (HG/PHG) for all three subclusters. As expected, these changes were also reflected in the findings with the whole VS as the seed.

In addition to the shared patterns of change, the daVS, dpVS, and vVS each showed specific alterations in connectivity. Moreover, the altered connectivities were associated with the clinical measures in CD. The strength of daVS connectivity with the lingual gyrus was positively correlated with amount of cocaine use in the prior month and cocaine craving. The vVS–vmPFC connectivity was negatively correlated with depression and anxiety. Together, the findings may provide new insight to delineating circuit level deficits in cocaine dependence. We highlighted some of the major findings in discussion.

### Decreased VS–hippocampus/parahippocampus (HG/PHG) rsFC in cocaine addiction

CD showed decreased connectivity of all three VS subergions with bilateral HG/PHG. The VS and HG/PHG are reciprocally connected as part of the limbic circuit^2,49^, and as with the VS, the hippocampus responds to cue-induced cocaine craving^7,50,51^. PET imaging reported cocaine cue-induced dopamine release in both HG and VS^52^. With fMRI, a double-blind study evaluated subjective effects of rush, high, low, and craving following cocaine (0.6 mg/kg) or saline infusion in cocaine addicted participants^9^. Cocaine-induced signal increases were observed in a host of brain regions including both HG and VS. Notably, activations of the HG, VS and some of the lateral prefrontal cortical regions are more correlated with craving than with rush ratings while other brain regions showed the opposite pattern of response.

In animal studies, cocaine decreased cerebral metabolism in both HG and VS in monkeys^53^. Unilateral infusion of N-methyl-d-aspartate in the HG increased dopamine efflux in the VS, and this effect was greater in rats receiving repeated cocaine as compared to controls receiving saline injections^49^. The results suggested that HG–VS circuit was impacted by repeated exposure to cocaine. Repeated cocaine exposure potentiated hippocampal inputs to the VS in in-vitro studies^54–56^. Further, following withdrawal from repeated cocaine administration, rats showed attenuated long-term potentiation in the HG–VS circuit^57^. Together, these studies suggest a critical role of the HG–VS circuit in supporting dopaminergic signaling and responses to cocaine craving. The current findings of decreased HG/PHG–VS connectivity may reflect an attenuated circuit activity in CD when they became abstinent recently. It would be of great interest to investigate whether diminished HG/PHG–VS connectivity may conduce to longer-term abstinence in a longitudinal study.

### VS-prefrontal cortical rsFC in cocaine addiction

As compared to HC, CD showed decreased functional connectivity between vVS and vmPFC, and the vVS–vmPFC connectivity was negatively correlated with depression and anxiety in CD. The vmPFC responds to drug cues in numerous imaging studies of substance abuse^58^. Anatomically and functionally interconnected^2^, the vmPFC and nucleus accumbens (NAc) are both implicated in the etiological processes of drug addiction^59^. For instance, vmPFC glutamatergic projections to the NAc were involved in reinstatement of cocaine seeking^60,61^. Increased Fos expression in the vmPFC, including those neurons projecting to NAc, was associated with context-induced reinstatement of heroin seeking^62^. In human imaging, transcranial direct current (in contrast with sham) stimulation of dlPFC enhanced the integrity of the white matter tracks connecting the vmPFC and NAc and alleviated drug craving^63^. Of direct relevance to the current finding, both the vmPFC and VS are involved in emotional regulation and the pathogenesis of depression^64^. Reduced gray matter volumes of both vmPFC and VS have been reported in depression, and deep brain stimulation may alter the activity of the vmPFC and VS in the treatment of depression^65^. A recent meta-analysis showed increased activities in both vmPFC and VS in relation to self-referential processing in depressed patients as compared with healthy controls^66^. Together with these earlier studies, the current findings suggest compromised vVS–vmPFC connectivity in association with negative mood and perhaps other emotion regulation deficits in cocaine dependence.

Notably, two earlier studies reported decreased^67^ and increased^19^ VS-vmPFC connectivity, respectively, in cocaine addiction, each seemingly in accord and in contrast with the current findings. However, the latter study employed ventral caudate as the seed region, whereas in the current work the vVS cluster is located at a more ventral location. In the former study, the vmFPC appeared to be in a location more ventral and posterior to the cluster we reported here. As with the VS, the vmPFC is known for subareal functional heterogeneity^68^. These disparities highlight the importance to carefully define functional subdivisions of an anatomical area.

We observed increased VS–MFC and dpVS–left IFC connectivity in CD. Studies have reported aberrant MFC/IFC activation during emotion processing in addicted individuals^58,69^. The VS responds to drug reward^70^ but also to negative emotions, noxious stimulation and pain^71–77^. In rodents, appetitive and defensive behavior is supported rostrocaudally in the NAc. Environmental manipulation (home vs. a new, overstimulated cage) can shrink or expand these response zones and prefrontal glutamatergic inputs were critical in determining the boundary^72^. Thus, increased VS–MFC/IFC connectivity may impact motivated behavior, perhaps in favor of overcharged negative responses, in CD. Further, the MFC/IFC is part of the task control circuit and disrupted in addicted individuals^42^. A previous study showed that the reduction in D2R signaling in the VS leads to reduced activity in the IFC^78^. Deactivations of both the VS and IFC were observed during decision making in relapsed cocaine users as compared to abstinent individuals^79^. Cocaine-induced craving correlated positively with activity in both VS and IFC during cocaine self-administration in non-treatment-seeking CD^8^. Increased MFC/IFC VS connectivity may be associated with deficits of affective and craving control in dependent cocaine users.

### VS-visual cortical connectivity in cocaine addiction

The daVS – visual cortex connectivity was positively correlated with the amount of cocaine use in the past month as well as the CCQ score. Although not typically a focus of the addiction literature, the visual cortex was often reported to be activated during exposure to drug cues^51,80,81^. A recent meta-analysis showed that 86% of published functional imaging studies of addiction reported significant drug cue-induced activity in the visual cortex^80^. Treatment-seeking participants as well as participants with strong motivation to quit demonstrated decreased cocaine cue-induced visual cortical activation, as compared to non-treatment-seeking and less motivated participants^82^. The current finding of daVS visual cortical connectivity in positive correlation with recent cocaine use and craving is consistent with this literature. Notably, changes in VS-visual cortical connectivity eluded analyses with the whole VS as a seed region.

### Other changes of VS rsFC in cocaine addiction

Both dpVS and vVS showed decreased rsFC with the hypothalamus in CD as compared to HC. The VS and hypothalamus both receive projections from the dopaminergic midbrain and respond to reward and motivated behavior^2^. Previous studies have implicated the hypothalamus in drug addiction^83,84^. In rodents, the transition from controlled to compulsive cocaine self-administration was associated with substantial remodeling of hypothalamic circuitry^85–87^. In humans, cocaine addicted individuals showed decreased hypothalamus activation viewing erotic vs. neutral pictures as compared to non-drug using controls^88^. Further, hypothalamus response to monetary reward vs. non-reward was negatively correlated with the duration of abstinence from cocaine use^89^. In both studies, the peak activities were located more in medial than in lateral hypothalamus (MNI coordinates: *x* = 0, *y* = 2, *z* = −14 and *x* = 3, *y* = 2, *z* = −14), consistent with an earlier report that the VS was more heavily connected to the medial than to lateral hypothalamus^90^. Decreased VS hypothalamus connectivity may have implications for altered motivational processes in cocaine addiction.

Compared to HC, CD showed decreased rsFC between daVS and cerebellum. Although the cerebellum is not conventionally considered as part of addiction circuit, it has been related to addiction by its conspicuous response to methylphenidate^91^. As the authors suggested, the cerebellar response could be explained as a downstream effect from dopaminergic stimulation of the striatum, as methylphenidate-induced increases in cerebellar activity is predicted by dopamine D2 receptor levels in striatum^91,92^. Although rarely discussed in detail, many studies showed cerebellar activation in association with cocaine administration or cue-induced cocaine craving^8,93–97^. More broadly, voxel-based morphometry^98–100^ showed decreased gray matter volume in the cerebellum in CD as compared to HC. In particular, decreases of gray matter in both striatum and cerebellum were associated with long-term exposure to cocaine^99^. The current findings add to this literature and research is needed to understand a more focused role of the cerebellum in addiction.

### Limitations of the study and conclusions

A few important limitations need to be considered. First, VS mapping based on connectivity differences builds on the rationale that rsFC's differ between CD and HC. Thus, the VS functional divisions as demonstrated here should be considered as specific to cocaine addiction. It remains to be seen whether these functional clusters apply to other substance use disorders or mental illnesses that implicate dopaminergic dysfunction. Second, the current work addressed rsFC and it remains unclear how the findings may apply to studies of cognitive and affective challenges in cocaine-dependent individuals. In summary, we parcellated the VS into three subclusters based on the differences in whole brain functional connectivity between CD and HC. The three VS subregions showed both shared and distinct patterns of altered connectivity. Changes in VS connectivity with the hippocampal and parahippocampal gyrus supports the importance of contextual memory in shaping habitual cocaine use; changes in VS connectivity with the visual cortex may reflect craving and drug seeking motivation; and changes in VS connectivity with the vmPFC are associated with depression and anxiety, a common characteristic of cocaine addicted individuals. Overall, the results confirm ventral striatal functional deficits in relation to cocaine misuse and provide more specific information about the subregional organization of these deficits.

## Electronic supplementary material

Supplementary text

Supplementary Figure 1
