# Supplementary material for: Ventral striatal dysfunction in cocaine dependence – difference mapping for subregional resting state functional connectivity
Source: Transl Psychiatry. 2018 Jun 18;8:119. doi: 10.1038/s41398-018-0164-0 (PMC6006289; doi:10.1038/s41398-018-0164-0)
Supplement: Supplementary file 1 — Supplementary text [file 41398_2018_164_MOESM1_ESM.docx]

### Supplementary Materials

Zhang and Li:

Ventral striatal dysfunction in cocaine dependence – Difference mapping for subregional resting state functional connectivity

**Supplementary Figure 1**: The distribution of 1000 best K values according to Bayesian Information Criterion (BIC).

**Supplementary Table 1**: Demographics of the subjects

| Subject characteristic | CD (n=66) | HC (n=66) | p-value |
| --- | --- | --- | --- |
| Ages (years) | 41.4 ± 7.3 | 39.3 ± 9.2 | 0.13* |
| Gender (M/F) | 44/22 | 36/30 | 0.34^ |
| Years of alcohol use | 14 ± 8.3 | 17 ± 10.3 | 0.05* |
| Amount of monthly cocaine use (gm) in the prior year | 31.4 ± 35.9 | N/A | N/A |
| Days of cocaine use in the prior month | 17.1 ± 9.5 | N/A | N/A |
| Years of cocaine use | 20.2 ± 6.9 | N/A | N/A |
| Cocaine craving questionnaire score | 23.8 ± 10.4 | N/A | N/A |

Note: values are mean ± S.D.; *two-tailed two-sample t test; ^$\chi^{2}$ test

**Supplementary Table 2:** Regions showing different functional connectivity between 66 CD and 66 HC.

| **volume** | **peak voxel** | **MNI coordinate** | | | **side** | **identified brain region** |
| --- | --- | --- | --- | --- | --- | --- |
| **(mm^3^)** | **(Z)** | **x** | **y** | **z** |  |  |
| *Dorsal anterior VS (CD > HC)* | | | | | | |
| 33,453 | 4.18 | -24 | 41 | 13 | L | Middle frontal gyrus |
|  | 4.16 | 30 | 56 | 7 | R | Middle frontal gyrus |
| ^3,375 | 4.05 | 27 | -67 | 4 | R | Lingual gyrus |
|  | 3.11 | 21 | -82 | 7 | R | Superior occipital gyrus |
| *Dorsal anterior VS (HC > CD)* | | | | | | |
| 24,705 | 4.75 | 30 | -25 | -14 | R | Hippocampal/Parahippocampal gyrus |
|  | 4.18 | -27 | -40 | -8 | L | Hippocampal/Parahippocampal gyrus |
| 7,965 | 4.53 | -15 | -49 | 19 | L | Precuneus |
|  | 3.99 | 12 | -40 | 28 | R | Posterior cingulate cortex |
| ^3,186 | 3.49 | -9 | -40 | -47 | L | Cerebellum |
| *Dorsal posterior VS (CD > HC)* | | | | | | |
| 8,586 | 4.64 | -45 | 17 | 25 | L | Inferior frontal cortex |
|  | 3.10 | -48 | -7 | 37 | L | Postcentral gyrus |
| 8,154 | 3.44 | -30 | 53 | 4 | L | Middle frontal gyrus |
| ^1,998 | 3.54 | -3 | 38 | 46 | L | Pre-supplementary motor area |
| ^1,242 | 4.21 | -12 | 2 | 73 | L | Supplementary motor area |
| *Dorsal posterior VS (HC > CD)* | | | | | | |
| 4,590 | 4.43 | 33 | -25 | -17 | R | Hippocampal/Parahippocampal gyrus |
| 5,427 | 3.99 | -30 | -40 | -8 | L | Hippocampal/Parahippocampal gyrus |
| 8,964 | 4.05 | -39 | 14 | -32 | L | Inferior Temporal gyrus |
| 4,617 | 3.75 | 39 | -4 | -35 | R | Inferior Temporal gyrus |
| ^3,186 | 3.84 | -15 | -46 | 13 | L | Precuneus |
| *486 | 2.98 | 0 | -2 | -14 | L/R | Hypothalamus |
| *Ventral VS (CD > HC)* | | | | | | |
| 7,020 | 3.82 | -27 | 41 | 19 | L | Middle frontal gyrus |
| *Ventral VS (HC > CD)* | | | | | | |
| ^5,481 | 4.47 | -27 | -34 | -8 | L | Hippocampal/Parahippocampal gyrus |
| ^4,239 | 4.17 | 24 | -34 | -8 | R | Hippocampal/Parahippocampal gyrus |
| ^864 | 3.04 | -9 | 41 | -8 | L | Ventromedial prefrontal cortex |
| *216 | 2.88 | 3 | 2 | -14 | L/R | Hypothalamus |

Note: voxel *p*<0.005 uncorrected and cluster-level *p*<0.05, FWE corrected (^ voxel p<0.005 uncorrected and AlphaSim p<0.05 corrected; * voxel p<0.005 uncorrected and cluster-level *p*<0.05 with small volume correction within the hypothalamus); R: right; L: left.
