# Supplementary figures and images for: Ventral striatal dysfunction in cocaine dependence – difference mapping for subregional resting state functional connectivity
Source: Transl Psychiatry. 2018 Jun 18;8:119. doi: 10.1038/s41398-018-0164-0 (PMC6006289; doi:10.1038/s41398-018-0164-0)

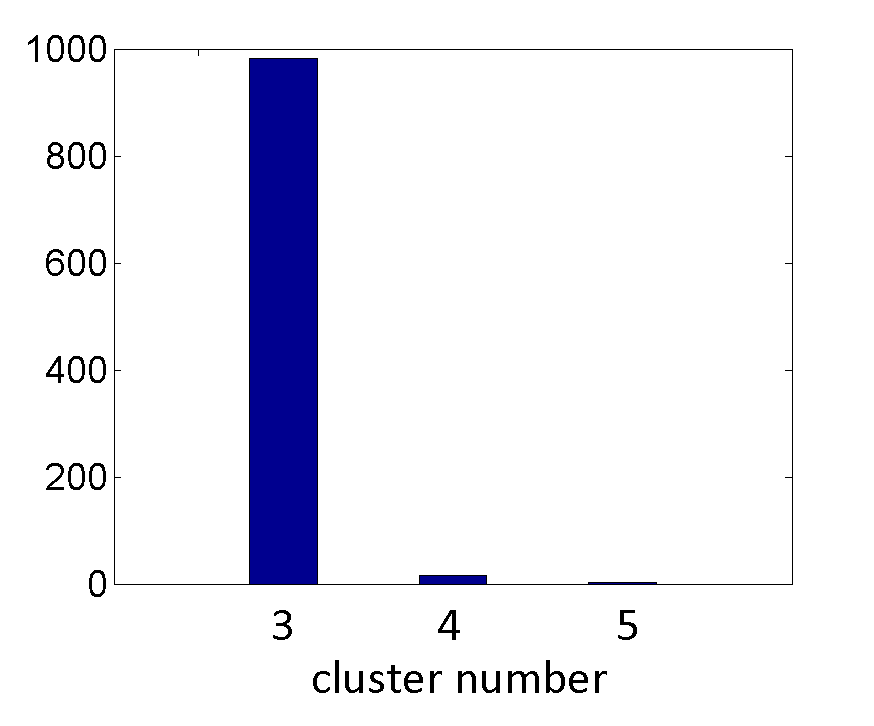

Supplement: Supplementary file 2 — Supplementary Figure 1 [file 41398_2018_164_MOESM2_ESM.tif]
